# Chemical composition, antioxidant, and antimicrobial activity of *Elsholtzia beddomei* C. B. Clarke ex Hook. f. essential oil

**DOI:** 10.1038/s41598-022-06358-6

**Published:** 2022-02-09

**Authors:** Teerapong Sripahco, Sarunpron Khruengsai, Rawiwan Charoensup, Jantrararuk Tovaranonte, Patcharee Pripdeevech

**Affiliations:** 1 School of Science, Mae Fah Luang University, Chiang Rai, 57100 Thailand; 2 Medicinal Plants Innovation Center, Mae Fah Luang University, Chiang Rai, 57100 Thailand; 3 School of Integrative Medicine, Mae Fah Luang University, Chiang Rai, 57100 Thailand; 4 Center of Chemical Innovation for Sustainability (CIS), Mae Fah Luang University, Chiang Rai, 57100 Thailand

**Keywords:** Biological techniques, Analytical chemistry, Secondary metabolism

## Abstract

The essential oil of *Elsholtzia beddomei* C. B. Clarke ex Hook. f. was investigated for its chemical composition and tested for antioxidant and antimicrobial activities. The *E. beddomei* essential oil was extracted using hydrodistillation for 4 h (yield of 1.38% w/w). Forty-three volatile compounds were identified in the *E. beddomei* essential oil, including linalool (83.67%), perillaldehyde (4.68%), neral (3.68%), perillene (1.65%), E-caryophyllene (1.55%), and α-zingiberene (1.06%) as the major compounds. The antioxidant activity of the *E. beddomei* essential oil was determined using 2,2-diphenyl-1-picrylhydrazyl (DPPH) free radical and 2,2-azino-bis(3-ethylbenzothiazoline-6-sulfonic acid (ABTS) radical cation scavenging activity. The IC_50_ values calculated using the DPPH and ABTS methods were 148.31 and 172.22 µg/mL, respectively. In addition, using disc diffusion and broth microdilution methods, the antimicrobial activities of the *E. beddomei* essential oil against *Escherichia coli*, *Pseudomonas aeruginosa*, *Enterobacter aerogenes*, *Staphylococcus aureus*, *Staphylococcus epidermidis*, *Bacillus subtilis*, and *Candida albicans* were evaluated. The *E. beddomei* essential oil possessed an inhibitory effect with the minimum inhibitory concentration in the range of 31.25–250.00 µg/mL among these pathogens. The results indicated that *E. beddomei* essential oil is an alternative raw material of food, and medicinal products for use in pharmaceutical applications.

## Introduction

The serious problem of antimicrobial resistance has been increased for public health threats due to rapid evolution and spread of microbial resistance especially clinically important bacterial species^1,2^. According to an increase of microbial resistance, several antimicrobial agents are losing their treating potential^1^. The therapeutic alternatives for treatments of the microbial infection may be limited or unavailable. In addition, World Health Organization (WHO) reported that infectious diseases have been evaluated as the second death cause around the world^3^. It was found that more than two million illnesses are caused from antimicrobial resistance with more than 20,000 deaths per year in the United States and these cases have been increased every year^1^. Therefore, searching of new alternative antimicrobial agents is necessary to decrease the microbial resistance problem.

Plants are evaluated as an enormous source of active secondary metabolites with medicinal properties^1^. These compounds are mainly employed by plant as a defense mechanism against plant pathogens, herbivores, and competitors^1^. The major secondary metabolites produced by plants are essential oils, phenolic compounds, alkaloids, lectins/polypeptides, and polyacetylenes^1^. Essential oils are complex volatile liquids containing a complex variety of terpenes and their derivatives. They are conventionally extracted using hydrodistillation, steam distillation, or mechanical processes^4^. The yield and composition of essential oil varies depending on ecological, onto genetic, climatic, post-harvest effects, and intra-species genetic factors^5,6^. Essential oils possess biological activities, including antibacterial, antiviral, antifungal, antitoxigenic, antiparasitic, and insecticidal activities^5^. They have also been reported to decrease the risks of diabetes, cancer, and cardiovascular diseases^7^. Therefore, researches on their use in several pharmaceutical, food, agricultural, and cosmetic industry applications have recently increased.

*Elsholtzia,* a genus of the family Lamiaceae, consists of at least 33 species. They are widely distributed in Europe, Africa, North America, and Asia, especially in China, Japan, Korea, and India in various cultivation conditions and altitudes^8^. Most *Elsholtzia* plants are aromatic and used as food, spices, herbal tea, beverages, folk medicine, cosmetics, andperfume^8^. Moreover, they have been used to treat colds, headaches, fever, pharyngitis, diarrhea, nephritises, rheumatic arthritis, and nyctalopia in China^8^.They have been evaluated to strongly inhibit the central nervous system and show an analgesic effect^9^. The essential oils of *Elsholtzia* species, including *E. cristata*, *E. blanda*, *E. bodinieri*, *E. ciliata*, *E. eriostachya*, *E. densa*, *E. rugulosa*, *E. ianthina*, *E. stauntonil*, and *E. splendens*, have been shown to possess antimicrobial activities^8^. *Elsholtzia* species also displayed other activities including antiviral activity (from *E. rugulosa* and *E. blanda*), antioxidant activity (from *E. splendens*, *E. rugulosa*, and *E. bodineri*), and anti-Alzheimer property (from *E. rugulosa*)^8^. Thus, the pharmacological use of the genus *Elsholtzia* have extensively increased.

Recently, *E. beddomei* C. B. Clarke ex Hook. f. and *E. stachyodes* (Link) Raiz. and Saxena have been discovered in Doi Hua Mod, Umpang, Tak province and in Doi Angkang, Fang, Chiang Mai province, respectively. Both plant species are evaluated as indigenous and aromatic plant species that are widely used as a local herb by hill tribes^10^.The essential oil of *E. stachyodes* has been described as an inhibitor of some bacterial strains, such as *Escherichia coli*, *Staphylococcus aureus*, and *Klebsiella pneumoniae*. Moreover, *E. stachyodes* oil also has antioxidant abilities as revealed by radical scavenging assay measurements and the β-carotene bleaching method^10^. In addition, carvacrol together with γ-terpinene have been found in the essential oil from aerial parts of *E. beddomei*^11^. It also possessed antibacterial activity against *S. aureus* and *Staphylococcus* epidermidis. However, less information is available on the chemical composition, antimicrobial activity, as well as antioxidant properties of *E. beddomei* essential oil. The purposes of this study are to identify the volatile compounds of the *E. beddomei* essential oil, and to determine its antioxidant activity based on the DPPH radical and ABTS radical cation scavenging activity.

## Results

### Chemical composition of *E. beddomei* essential oil

**Figure 1:**
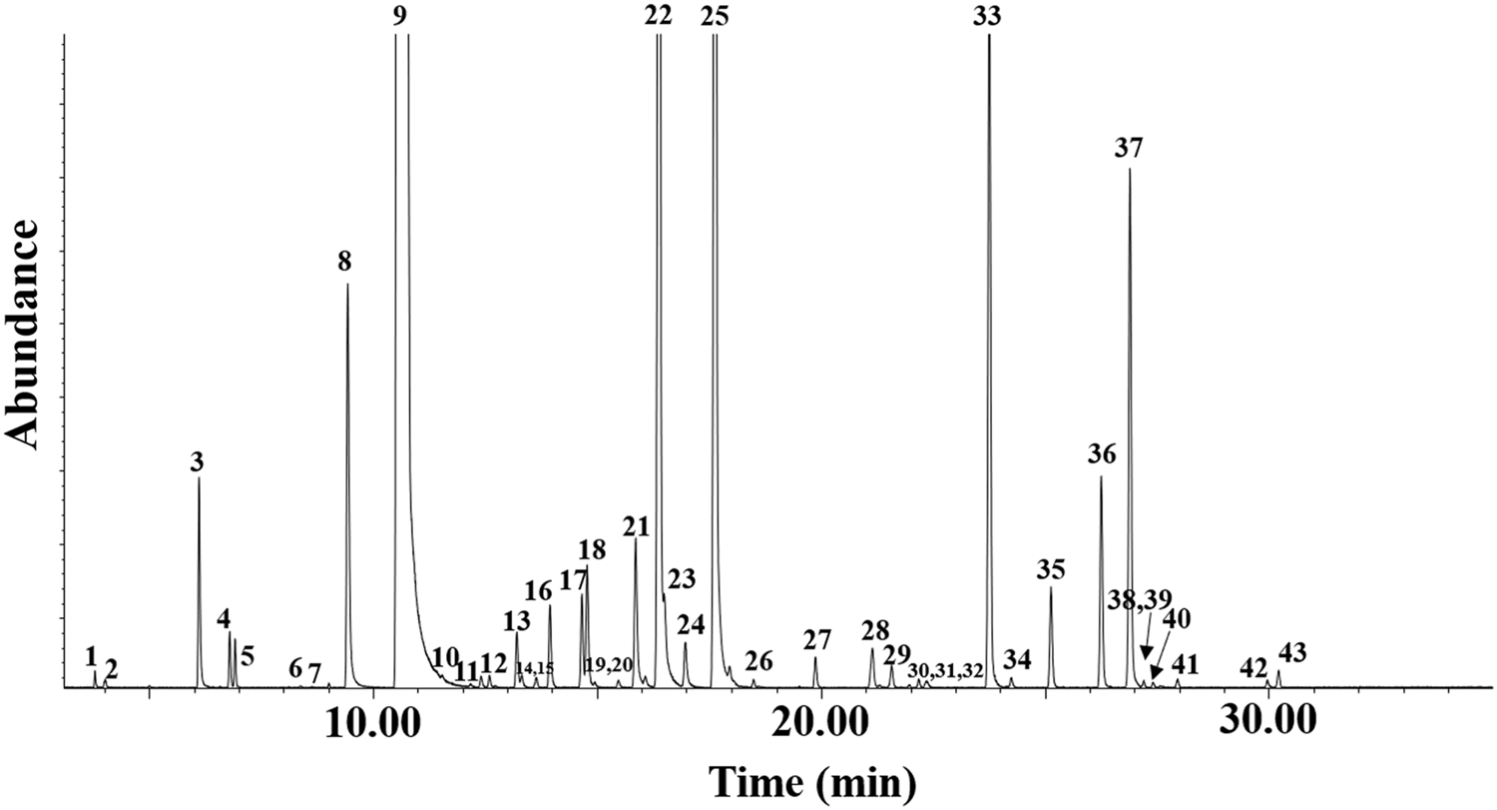
GC–MS total ion current chromatogram of *E. beddomei* essential oil with identified peaks listed in Table 1.

The hydrodistillation of the *E. beddomei* essential oil provided a yield of 1.38% v/w. The volatile compounds of the *E. beddomei* essential oil are shown in Fig. 1 and Table 1. In total, 43 volatile compounds were identified, representing 100% of the oil. Linalool (83.67%), perillaldehyde (4.68%), neral (3.68%), perillene (1.65%), E-caryophyllene (1.55%), and α-zingiberene (1.06%) were the major volatile compounds of the *E. beddomei* essential oil. These results showed a high content of monoterpene hydrocarbons (over 95%) in *E. beddomei* essential oil.

**Table 1:** Chemical constituents of *E. beddomei* essential oil.

No	Compound	RI^a^	%Peak area
1	2E-hexenal	854	t^b^
2	hexanol	872	t
3	benzaldehyde	962	0.31
4	endo-2-norborneol	991	0.07
5	cis-meta-mentha-2,8-diene	993	0.06
6	benzyl alcohol	1036	t
7	bergamal	1059	t
8	acetophenone	1070	0.81
9	linalool	1106	83.67
10	perillene	1113	1.65
11	exo-isocitral	1151	t
12	trans-α-necrodol	1155	t
13	β-pinene oxide	1166	0.12
14	lavandulol	1177	t
15	rosefuran epoxide	1185	t
16	Z-isocitral	1189	0.15
17	verbanol	1210	0.16
18	elsholtzia ketone	1214	0.24
19	trans-pulegol	1225	t
20	nerol	1239	0.33
21	exo-fenchyl acetate	1241	t
22	neral	1247	3.68
23	trans-chrysanthenyl acetate	1247	0.26
24	geraniol	1261	0.11
25	perillaldehyde	1282	4.68
26	nerylformate	1293	t
27	methyl geranate	1335	0.06
28	eugenol	1370	t
29	neryl acetate	1373	0.05
30	α-ylangene	1387	t
31	geranyl acetate	1393	t
32	p-but-E-enyl-anisole	1394	t
33	E-caryophyllene	1431	1.55
34	α-trans-bergamotene	1446	t
35	α-humulene	1467	0.23
36	γ-muurolene	1493	0.42
37	α-zingiberene	1508	1.06
38	aciphyllene	1516	t
39	E,E-α-farnesene	1520	t
40	γ-cadinene	1528	t
41	δ-cadinene	1537	t
42	longipinanol	1583	t
43	himachalene epoxide	1594	t
	Total		100

^a^Calculated retention indices obtained from a DB-5 column.

^b^Trace amount < 0.05.

### Antioxidant activity

The antioxidant activities of *E. beddomei* essential oil using the DPPH and ABTS scavenging assays are shown as IC_50_ values in Table 2 and compared to those obtained from trolox, linalool, and perillaldehyde. The IC_50_ of *E. beddomei* essential oil was measured to be 148.31 ± 0.23 µg/mL and 172.22 ± 0.32 µg/mL using the DPPH and ABTS assays, respectively. Trolox showed significantly higher antioxidant activity with IC_50_ of 7.34 ± 0.12 µg/mL and 11.31 ± 0.15 µg/mL from DPPH and ABTS assays, respectively. Linalool and perillaldehyde, the major components of *E. beddomei* essential oil, also showed significant lower antioxidant activity than those of *E. beddomei* essential oil with IC_50_ of 201.23 ± 0.26 µg/mL and 216.28 ± 0.18 µg/mL, and 186.43 ± 0.27 µg/mL and 197.61 ± 0.63 µg/mL using the DPPH and ABTS assays, respectively.

**Table 2:** Antioxidant activities of essential oil and standards.

Sample	Yield	IC_50_ (µg/mL)	
	(%w/w)	DPPH	ABTS
*E. beddomei* essential oil	1.38 ± 0.21	148.31 ± 0.23^b^	172.22 ± 0.32^b^
trolox	-	7.34 ± 0.12^a^	11.31 ± 0.15^a^
linalool		201.23 ± 0.26^d^	226.28 ± 0.18^d^
perillaldehyde		186.43 ± 0.27^c^	197.61 ± 0.63^c^

The data are mean ± standard deviation. Different letters indicate significant differences (*p* < 0.05).

### Antimicrobial activity

The antimicrobial activity of the *E. beddomei* essential oil against seven pathogenic microorganisms was evaluated using disc diffusion and broth microdilution methods. The zone of inhibition diameter, minimum inhibitory concentration (MIC), and minimum microbicidal (MMC) of the *E. beddomei* essential oil and chloramphenicol are shown in Table 3. It was found that Gram-positive and Gram-negative bacteria, and *C. albicans* were suppressed by the *E. beddomei* essential oil. The results from the disc diffusion method indicated that Gram-positive bacteria were the most significantly sensitive to 10 µg/mL of *E. beddomei* essential oil with a zone diameter ranging between 25.17 and 29.91 mm, compared to those obtained from other tested pathogens ranging between 19.34 and 22.23 mm. The MIC of the bacterial pathogens ranged from 3.91 to 7.81 µg/mL, whereas the MIC for *C. albicans* was 15.62 µg/mL. The MIC values of chloramphenicol were 31.25–125 µg/mL and 125 µg/mL for bacterial pathogens and *C. albicans*, respectively. The results of the MMC determination indicated that the *E. beddomei* essential oil had the lowest MMC value (3.91 µg/mL) for all Gram-positive bacteria.

**Table 3:** Antimicrobial activity of *E. beddomei* essential oil and chloramphenicol.

Microorganism	Chloramphenicol			*E. beddomei* essential oil		
	MIC (µg/mL)	MMC (µg/mL)	Zone of inhibition diameter (mm)	MIC (µg/mL)	MMC (µg/mL)	Zone of inhibition diameter (mm)
**Gram-positive bacteria**						
*S. aureus* ATCC 25923	3.91^a^	15.62^a^	35.13 ± 1.44^a^	3.91^a^	31.25^b^	25.17 ± 0. 23^b^
*S. epidermidis* ATCC 12228	3.91^a^	15.62^a^	34.94 ± 0.81^a^	3.91^a^	15.62^a^	29.75 ± 0.21^a^
*B. subtilis* ATCC 6051	3.91^a^	15.62^a^	34.88 ± 1.21^a^	3.91^a^	15.62^a^	29.91 ± 0.20^a^
**Gram-negative bacteria**						
*E. coli* ATCC 25922	7.81^b^	62.50^c^	28.91 ± 0.52^c^	7.81^b^	62.50^c^	21.86 ± 0.20^c^
*P. aeruginosa* ATCC 27853	3.91^a^	31.25^b^	32.24 ± 0.52^b^	7.81^b^	62.50^c^	22.23 ± 0.31^c^
*E. aerogenes* ATCC 13048	7.81^b^	62.50^c^	28.81 ± 0.48^c^	7.81^b^	62.50^c^	21.18 ± 0.25^c^
**Fungus**						
*C. albicans* ATCC 10231	7.81^b^	62.50^c^	28.01 ± 0.21^c^	15.62^c^	125.00^d^	19.34 ± 0.30^d^

The data are mean ± standard deviation. Different letters indicate significant differences (*p* < 0.05).

## Discussion

The obtained results on the chemical composition of essential oils extracted from *Elsholtzia* plants species differed from previous studies. Previously, α-pinene and β-pinene were evaluated as the major volatile compounds in at least 15 species of *Elsholtzia* plants^8,12^. The essential oils of *E. rugusola* and *E. splendens* revealed thymol and carvacrol as major compounds^13,14^, whereas *E. ciliata* and *E. patrini* essential oils showed high content of β-dehydroelsholtzia ketone and elsholtzia ketone^15^. It was indicated that species have specific volatile profiles. Moreover, carvacrol and γ-terpinenewere found as the main compound in *E. beddomei* essential oil reported by Phetsang et al.^11^ which was different from this study. Plant age, geography, day length, temperature, cultivation, climatic conditions, selection of plant organ, and harvest period, as well as extraction method, may influence the essential oil composition. These factors may result in the biosynthesis pathways of the plant and, consequently, the chemical composition and content, leading to the production of diverse chemotypes^16,17^. Similarly, the variety of essential oil compositions of *Elsholtzia* species was reported in previous studies. Fang et al.^18^ and Ren et al.^14^ described that the content of α-pinene in the essential oil of *E. blanda* growing in Yunnan province in China was4.84% while those cultivated in Sichuan province in China was 1.43%. In addition, the volatile components of the essential oil from *E. stauntonii* obtained from different extraction methods significantly varied in terms of content and type^19,20^.

The potential of essential oil for free radical inhibition may involve the specific compounds eliminating and preventing free radical formation^21,22^. Several studies on the antioxidant activity of extracts obtained from *Elsholtzia* species have been previously reported. High antioxidant activity was found in the extracts from *E. ciliata*^23^, *E. rugulosa*^24^, *E. bodineri*^25^, *E. ciliata*obtained from CO_2_ supercritical fluid extraction^26^, *E. splendens*^27^, and *E. blanda*^28^. Although the antioxidant activity of *Elsholtzia* plants has been studied extensively, the antioxidant activity of the *E. beddomei* essential oil have not been reported yet. The antioxidant activity of *E. beddomei* essential oil may be influenced by non-phenolic constituents such as monoterpenic compounds and its derivatives. The combination of major and minor compounds in the essential oil may enhance antioxidant activity via the synergistic effects among these compounds producing an effective defense system against free radical attack. Several previous studies also confirmed linalool as a valuable antioxidant compound with promising nutraceutical applications due to its donation of hydrogen atoms and removing the electron from DPPH^29,30^. Perillaldehyde was also evaluated mainly as an antioxidant compound of essential oil of *Perilla frutescens* (L.) Britton inhibiting inflammatory skin diseases or disorders related to oxidative stress^31–33^. The antioxidant mechanism of perillaldehyde has been found by inhibiting BaP-induced AHR activation and ROS production and BaP/AHR-mediated release of the CCL2 chemokine while activating the NRF2/ HO1 antioxidant pathway^40^. Other compounds, such as neral, E-caryophyllene, and α-zingiberene, were reported to exhibit antioxidant activity due to the activated methylene group in their chemical structures^34^.

The antibacterial activity of essential oils of *Elsholtzia* plants against pathogenic bacteria has been previously investigated. Essential oils of *E. splendens* showed antibacterial inhibitory effects against *S. aureus*, *S. epidermidis*, and *Propionibacterium acnes*^35^. Similarly, *E. ciliata* and *E. rugulosa* essential oils also inhibited *S. aureus*, *P. aeruginosa*, *Bacillus enteritidis*, *B. subtilis*, *Proteus vularis*, *Shigella dysenteriae*, and *E. coli*^36,37^.Essential oils from several *Elsholtzia* species revealed extensive antibacterial activity against some bacteria involved inhuman respiratory infections such as *Aeruginosus bacillus* and *Diplococcus intracellularis*^31,32^. Moreover, the *E. blanda* and *E. rugulosa* essential oils showed significant inhibitory activity against methicillin resistant *S. aureus* strain^33^. A report on the antibacterial activity of essential oil extracted from *E. beddomei* was found in the study of Phetsang et al.^11^ *E. beddomei* displayed an inhibitory effect against *S. aureus* and *S. epidermidis*.

The antimicrobial activity of *E. beddomei* essential oil may be correlated mainly with the presence of active components, including monoterpenes, sesquiterpenes, and their derivatives, as reported by Burt^5^. The major active compound enhancing the antimicrobial property of *E. beddomei* essential oil is linalool, which is abundant in thyme essential oils and has been reported to show antimicrobial properties^5^. In addition, the significant antimicrobial activity of *E. beddomei* essential oil could result from the synergetic effects of the essential oil composition^36,37^. Various studies proposed that the mechanism of action of monoterpenes and its derivatives was membrane permeability, based on their ability to disrupt the cell wall and cytoplasmic membrane resulting in lysis and leakage of intracellular components^5,37^. The interaction of the antimicrobial compounds of the essential oil with the membrane can disturb the transportation of nutrients and ions, the membrane potential, and the overall cell permeability^47^. However, there is limited information about the in vivo antimicrobial activity mechanism of *E. beddomei* essential oil. Thus, in vivo experiments should be improved in order to apply *E. beddomei* essential oil to the best of its functional potential. In addition, toxicological and regulatory investigations are also necessary before using *E. beddomei* essential oil as a biological agent.

## Conclusion

The major volatile components of the *E. beddomei* essential oil were linalool, perillaldehyde, neral, perillene, E-caryophyllene, and α-zingiberene. The essential oil of *E. beddomei* showed antioxidant activity as determined by DPPH and ABTS assays. It also showed inhibitory effects against Gram-negative and Gram-positive bacteria, as well as fungus *C. albicans*, based on the presence of linalool. These findings demonstrate that *E. beddomei* essential oil may be an alternative natural product and a potential source of pharmaceutical agent. Toxicological and clinical tests are further needed before applying it in humans.

## Materials and methods

### Plant material

The aerial parts of *E. beddomei* were collected from Doi Hua Mod, Umpang, Tak Province, Thailand in July 2020. This plant species is not endangered. It was identified by taxonomist Dr. Jantrararuk Tovaranonte and the voucher specimen MFU 10,232 was deposited at the Mae Fah Luang Botanical Garden, Mae Fah Luang University, Chiang Rai, Thailand. The specimen was collected in the field with permission from the Department of National Parks, Wildlife and Plant Conservation, Umpang, Tak Province, Thailand. The study on this plant species has comply with relevant institutional, national, and international guidelines and legislation.

### Essential oil extraction

The fresh aerial parts of *E. beddomei* were subjected to hydrodistillation using a Clevenger-type apparatus for 4 h. The obtained essential oil was dried with anhydrous sodium sulfate to eliminate the water. The essential oil was kept in a sealed vial and stored at 4 °C for further use.

### Identification of chemical composition by gas chromatography-mass spectrometry (GC–MS)

A total of 0.5% v/v essential oil was prepared by diluting with dichloromethane. A total of 1.0 μL of solution was injected into the gas chromatography-mass spectrometry (GC–MS) using an Agilent 6890 N gas chromatograph (Agilent Technologies, Santa Clara, CA, USA) equipped with electron impact ionization and mass-selective detector (Agilent 5973, Agilent Technologies, Santa Clara, CA, USA). A fused-silica capillary DB5-MS (30 m × 0.25 mm i.d., 0.25 µm) (J&W Scientific, USA) was used with helium, a carrier gas, with a rate of 1.0 mL/min. The injector temperature was set at 250 °C. The ionization energy was 70 eV in electron impact ionization mode with an ion source temperature of 250 °C and interface temperature of 250 °C. The oven temperature program was initiated at 60 °C and then increased to 240 °C with a rate of 3 °C/min. The acquisition was performed in scan mode (m/z 30–300). The volatile components were identified using the computer matching method and by comparing the mass spectra with the Wiley 7 N and W8N08 libraries. The Kovats retention indices were calculated using linear interpolation of the retention times of C_9_–C_16_ n-alkanes and were compared with the relevant literature reported by Adams^38^ and the mass spectra from the Wiley 7 N library. Semi-quantitative analysis (given as % peak area of the particular component) was determined by area normalization method. Calculation of area percent is assumed to be equal to weight percent. The %area calculation procedure reports the area of each peak in the run as a percentage of the total area of all peaks in the run. Area percentage does not require prior calibration and does not depend upon the amount of sample injected within the limits of the detector so response factors were not used.

### Antioxidant activity

#### DPPH assay

The DPPH radical was used in the antioxidant investigation of *E. beddomei* essential oil, similar to the method reported by Insawang et al.^39^ A 60 µM DPPH solution was prepared in methanol. The essential oil was diluted in 10% dimethyl sulfoxide (DMSO) in concentrations of 1000, 500, 250, 125, 62.50, 31.25, 15.62, 7.81 and 3.91 µg/mL. A mixture consisting of 50 µL of essential oil or standard solutions and 1950 µL of DPPH solution was then made and the reaction was monitored in the dark at room temperature. The solution was then incubated for 30 min. The absorbance of the mixture was determined at 517 nm using a PerkinElmer spectrophotometer. Methanol was used as a blank solution. The scavenging capacity were calculated using following equation.

$${(A}_{C}-{A}_{S}/{A}_{C})\times 100$$

where ${A}_{C}$ and ${A}_{S}$ correspond to the absorbance of the control and sample, respectively. The antioxidant activity of *E. beddomei* essential oil, trolox, linalool and perillaldehyde were reported as IC_50_. Each sample was tested for antioxidant activity in triplicate.

#### ABTS assay

The scavenging activity of *E. beddomei* essential oil against ABTS was investigated in a manner similar to Insawang et al.^39^ The ABTS radical cation reagent was prepared by combining 7 mM ABTS solution with 2.45 mM potassium persulfate. The reagent was kept in the dark at room temperature. The *E. beddomei* solution was prepared in methanol (methanol concentration = 1000, 500, 250, 125, 62.50, 31.25, 15.62, 7.81, and 3.91 µg/mL). For each sample, a reaction was prepared by mixing 50 µL of the essential oil or standard solutions and 1950 µL of the ABTS solution before shaking vigorously and then kept in the dark at room temperature for 30 min. The absorbance of the solution was determined as 734 nm using a PerkinElmer spectrophotometer. Methanol was used as a blank solution. The scavenging capacity was calculated using the equation described above. The antioxidant activity of *E. beddomei* essential oil, trolox, linalool and perillaldehyde were reported as IC_50_. Each sample was tested for antioxidant activity in triplicate.

### Antimicrobial activity

#### Microbial strains

The antimicrobial activity of *E. beddomei* essential oil against seven human pathogens (*S. aureus* ATCC 25923, *S. epidermidis* ATCC 12228, *B. subtilis* ATCC 9372, *E. coli* ATCC 25922, *P. aeruginosa* ATCC 27853, *E. aerogenes* ATCC 13048, and fungus *C. albicans* ATCC 10231) was measured. These pathogens were obtained from the culture collection of the Thailand Institute of Scientific and Technological Research, Bangkok, Thailand.

#### Disc diffusion method

The antimicrobial activity of *E. beddomei* essential oil was screened by the disc diffusion method according to Lu et al.^40^ with some modifications. All bacterial pathogens were cultured in Mueller Hinton agar while *C. albicans* was cultured in potato dextrose agar (PDA). All bacterial and fungal pathogens were then sub-cultured in nutrient broth and potato dextrose broth (PDB), respectively. The bacterial pathogens were incubated at 37 °C for 24 h while the fungus pathogen was incubated at 28 °C for 48 h. The active bacterial pathogens were prepared in a nutrient broth until it matched the 0.5 McFarlane standard (1 × 10^8^ colony-forming unit/mL). The bacterial pathogens were spread out on the dried surface of the nutrient agar plates. The serialized 6 mm diameter paper discs (Whatman, USA) were impregnated with 30 µL of *E. beddomei* essential oil solution (10 µg/mL) preparing in 10% DMSO. The paper discs were then placed on the nutrient agar plate. These plates were incubated at 37 °C for 24 h. The fungus plates were incubated at 28 °C for 48 h. The diameter of the zone inhibition was determined. Chloramphenicol (10 µg/mL) and 10% DMSO were used as the positive and negative controls, respectively. Each experiment was performed in replicates.

#### Determination of MIC and MMC

The MIC and MMC were determined according to the modifications of Teh et al.^41^ The *E. beddomei* essential oil was prepared in 10% DMSO. The dilution series of *E. beddomei* essential oil were performed by two-fold dilution in concentrations of 1000, 500, 250, 125, 62.50, 31.25, 15.62, 7.81, and 3.91 µg/mL. The reaction was achieved by mixing 50 µL of essential oil, 10 µL of microbial suspension, and 10 µL of 0.675% of resazurin (Sigma-Aldrich, USA) on a 96-well microtiter plate. Chloramphenicol and 10% DMSO as the positive and negative controls, respectively. The bacterial and fungal plates were then incubated at 37 °C for 4.5 h and 28 °C for 48 h, respectively. The MIC was determined by considering the pink color of resazurin. Furthermore, MMC was also determined by mixing the solution from the well with a violet color of resazurin and spreading it on nutrient agar or PDA plate prior to incubation at 37 °C for 24 h and 28 °C for 48 h for bacteria and fungus, respectively. The MMC was determined by evaluating the plates without microbial colony. Each experiment was performed in triplicate.

### Statistical analysis

Results are expressed as mean ± standard deviation. All experiments were performed in triplicate. Analysis of variance (ANOVA) was performed to measure the antimicrobial and antioxidant activities. The mean comparison was based on the Student's t-test at *p* < 0.05. All statistical tests were performed using the SPSS statistics software (IBM SPSS Statistics for Windows, Version 22.0. Armonk, NY, IBM Corp).
